# Prevalence and risk factors of herpes zoster in patients with rheumatoid arthritis: a systematic review and meta-analysis

**DOI:** 10.3389/fimmu.2026.1754915

**Published:** 2026-05-08

**Authors:** Shuo Wang, Qiang Liu, Peng Zhou, Wenfeng Hu, JunHu Liu

**Affiliations:** 1 Hubei University of Chinese Medicine, Wuhan, China; 2 Xianning Chinese Medicine Hospital, Xianning, Hubei, China; 3 Department of Rehabilitation Medicine and Physical Therapy, Hubei Provincial Corps Hospital, Chinese People’s Armed Police Forces, Wuhan, Hubei, China

**Keywords:** rheumatoid arthritis, herpes zoster, risk factors, disease burden, immunosuppression, opportunistic infections, shingles, varicella-zoster virus

## Abstract

**Background:**

The co-occurrence of herpes zoster (HZ) in patients with rheumatoid arthritis (RA) represents a significant public health concern, with notable implications for both physical and mental health. This meta-analysis aims to systematically evaluate the pooled proportion of HZ in RA patients and identify associated factors.

**Methods:**

A comprehensive literature search was conducted across eight databases: PubMed, Web of Science, Embase, Cochrane Library, CNKI, VIP, WANFANG Data, and CBM. The review was conducted in accordance with PRISMA guidelines, and the quality of included studies was assessed using the Newcastle-Ottawa Scale (NOS). All statistical analyses were performed using Stata 17.0.

**Results:**

A total of 17 observational studies (seven case-control and ten cohort) comprising 472,150 patients were included in this meta-analysis, indicating a descriptive pooled proportion of herpes zoster of 6% (95% CI: 4%-8%). However, the 95% prediction interval was 5% to 45%, reflecting substantial heterogeneity. This estimate represents an average across diverse settings rather than a universally generalizable figure. Eleven potential factors were evaluated, and the results indicated that the following were significantly associated with HZ in RA patients: female gender (OR = 1.47, 95% CI, 1.15-1.89, P = 0.002), age (OR = 1.12, 95% CI, 1.02-1.22, P = 0.012), corticosteroid dosage ≥7.5 mg/day (OR = 2.16, 95% CI, 1.85-2.53, P < 0.001), corticosteroid use (OR = 1.42, 95% CI, 1.19-1.69, P < 0.001), use of tumor necrosis associated factor inhibitors (OR = 1.94, 95% CI, 1.43-2.63, P < 0.001), methotrexate use (OR = 1.68, 95% CI, 1.39-2.02, P < 0.001), hydroxychloroquine use (OR = 2.67, 95% CI, 1.24-5.74, P = 0.012), history of pulmonary disease (OR = 1.42, 95% CI, 1.10-1.83, P = 0.007), history of hypertension (OR = 1.43, 95% CI, 1.15-1.77, P = 0.001), history of kidney disease (OR = 1.30, 95% CI, 1.04-1.62, P = 0.022), and history of heart disease (OR = 2.30, 95% CI, 1.17-4.52, P = 0.016).

**Conclusion:**

Our meta-analysis indicates that the observed 6% pooled proportion of HZ in patients with RA constitutes a significant burden compared to the general population, highlighting the necessity for timely prevention. Moreover, when assessing HZ risk, factors such as female gender, age, corticosteroid use and dosage ≥7.5 mg/day, use of tumor necrosis factor inhibitors, methotrexate, hydroxychloroquine, and a history of pulmonary disease, hypertension, kidney disease, or heart disease should be carefully considered. These findings highlight the need for further research into the associated factors and underlying biological mechanisms of HZ in RA patients and support the development of targeted prevention strategies that address modifiable risks.

**Systematic Review Registration:**

https://www.crd.york.ac.uk/PROSPERO/view/, identifier CRD420251050627.

## Introduction

Herpes zoster (HZ) is a common disease caused by reactivation of the latent varicella zoster virus (VZV) in the dorsal root ganglia. Reactivation can occur due to immune decline or immunosenescence, leading to recurrent episodes (1). HZ significantly impairs patients’ quality of life, causing complications such as post-herpetic neuralgia (PHN), vascular disorders, and serious neurological or ophthalmological conditions (2). These complications often increase medical costs and further reduce quality of life (3). Data from the Consortium of Rheumatology Researchers of North America registry indicate that VZV infection is the most frequent opportunistic infection among patients with RA (4), and studies in Asia have similarly reported an elevated risk of HZ in RA patients (5). RA is a chronic autoimmune disease characterized by joint inflammation, synovitis, morning stiffness, and/or limited mobility of the proximal interphalangeal joints (6). Immunocompromised individuals, including those with RA, are at higher risk of VZV infection. Research confirms a close association between RA and HZ (4). RA is also linked to increased mortality and reduced quality of life (7), as it is a systemic autoimmune disorder that can cause progressive disability and premature death (8). When RA patients are co-infected with VZV, resulting in HZ, the clinical situation is further exacerbated. Therefore, preventing or minimizing the occurrence of HZ is of critical importance for individuals with RA.

Studies (9, 10) indicate that patients with RA have an increased risk of developing HZ. Although the global prevalence of RA is less than 1% (11), among the known risk factors for HZ, RA demonstrates the highest odds ratio, ranging from 1.37 to 1.57 across all age groups (12). Furthermore, the prevalence of HZ is rising (13), accompanied by substantial morbidity. While the absolute proportion may appear modest, it represents a significantly elevated risk compared to the general population, underscoring that the impact of HZ on RA patients must not be overlooked. Given their elevated HZ risk, vaccination is a crucial preventive strategy for RA patients. Historically, live-attenuated vaccines were restricted in immunocompromised individuals due to safety concerns (14). However, the recombinant zoster vaccine (RZV) provides a safer and effective alternative. Despite a slight risk of baseline disease flare-ups, RZV exhibits a favorable safety profile and effectively prevents severe HZ, strongly supporting its integration into tailored vaccination programs (15).The latest meta-analysis (16) confirms that RA patients have a significantly elevated risk of HZ, with particularly higher incidence observed in the 50–60 age group compared to healthy elderly individuals (10). The development of HZ results from the interaction of multiple factors (17); however, a systematic investigation into the reasons for its co-occurrence in RA patients is still lacking. Therefore, the present study aims to systematically evaluate the pooled proportion of HZ and identify associated factors in the broad adult RA population, encompassing patients from diverse clinical settings. By synthesizing data across different treatment backgrounds and healthcare contexts, this meta-analysis seeks to provide a descriptive summary of the reported proportions of HZ to inform targeted preventive strategies.

## Methods

The protocol for this systematic review was registered in the International Prospective Register of Ongoing Systematic Reviews (PROSPERO registration ID: CRD420251050627) on May 11, 2025. The review was conducted in accordance with the updated Preferred Reporting Items for Systematic Reviews and Meta-Analyses (PRISMA) guidelines (18).

### Search strategy

A comprehensive literature search was performed across eight databases: PubMed, Web of Science, Embase, Cochrane Library, China National Knowledge Infrastructure (CNKI), Weipu Database (VIP), Wanfang Database (WANFANG), and the Chinese Biomedical Database (CBM), covering all records up to May 2025. Both Medical Subject Headings (MeSH) and free-text terms were used in combination. Additionally, reference lists of included studies and relevant reviews were manually screened to identify any further eligible publications. A detailed description of the search strategy is provided in **Table 1** of the **Supplementary Materials**.

### Study selection and inclusion criteria

This study strictly followed the PICOS principle to formulate the inclusion and exclusion criteria. Population: Adult patients with a confirmed diagnosis of rheumatoid arthritis were included, regardless of treatment setting, to ensure a comprehensive estimation. Intervention: RA patients that exhibited HZ. Comparison: RA patients that didn’t exhibit HZ. Outcome: RA patients were complicated with HZ and related measurement data. Study design: To ensure a comprehensive evaluation of the burden of disease, observational studies including cohort, case-control, and cross-sectional designs were considered eligible for inclusion. Exclusion criteria included studies without full text, unpublished reports, abstracts, editorials, irretrievable studies, letters, qualitative studies, and studies that did not report outcomes related to HZ.

### Data extraction

Two researchers independently screened the literature and extracted relevant data based on the predefined inclusion and exclusion criteria. Discrepancies were resolved through consultation with a third researcher. When essential information was missing from the original studies, the corresponding authors were contacted via email. Extracted data included the first author, publication year, study design, country, sample size, regression model, and identified influencing factors.

### Quality appraisal

The quality of included studies was independently assessed by two researchers using the Newcastle-Ottawa Scale (NOS) (19). The NOS evaluates observational studies across three domains selection, comparability, and outcome on a 9-point scale. The selection domain considers population representativeness, comparability between exposed and non-exposed groups, confirmation of exposure, and absence of outcome at baseline. The comparability domain assesses the control of confounding factors, while the outcome domain evaluates outcome assessment independence, follow-up duration, and completeness. Any scoring discrepancies were resolved by a third reviewer. Studies scoring ≥7 points were considered high quality, those scoring 4–6 points were classified as moderate quality, and studies scoring ≤3 points were excluded from the analysis.

### Statistical analyses

Statistical analyses Stata 17.0 and R software (using the ‘meta’ package) were performed to conduct the statistical analyses. Specifically, the number of HZ cases and the total number of RA patients were extracted from each study to calculate the proportion. For the pooled proportion analysis, the Freeman-Tukey double arcsine transformation was applied to stabilize variances. A random-effects model with the DerSimonian-Laird estimator was employed, and a 95% prediction interval was calculated to estimate the range of the proportion in future studies, accounting for between-study heterogeneity. Data from different study designs were harmonized by treating them as single-arm proportions based on the reported sample sizes. The calculation of the prediction interval relies on the assumption that the random effects follow a normal distribution. The selection of variables for the single-factor meta-analysis was determined by data availability. Potential risk factors were included in the quantitative synthesis only if extractable data (effect sizes and 95% CIs) were reported by at least three independent studies. Variables reported by fewer than three studies were excluded from the pooled analysis to ensure statistical reliability. Continuous variables were presented as weighted mean differences (WMD) with 95% confidence intervals (CI), while dichotomous variables were expressed as odds ratios (OR). The sign of the WMD indicates the direction of effect, and its magnitude reflects the clinical impact. An OR > 1 indicates an increased risk. Heterogeneity among studies was assessed using the P value and I² statistic. Heterogeneity among studies was evaluated using the P value and I^2^ statistic. A fixed-effects model was applied when heterogeneity was considered negligible (P≥ 0.10 and I^2^ < 50%). Conversely, a random-effects model was adopted when significant heterogeneity was observed (P < 0.10 or I^2^≥ 50%). To assess the robustness and reliability of the pooled results, a ‘leave-one-out’ sensitivity analysis was performed. This method involved iteratively omitting one study at a time from the meta-analysis and recalculating the pooled effect size to determine if any single study exerted a disproportionate influence on the overall estimate. Subgroup analyses were conducted to explore potential sources of heterogeneity based on publication year (before 2010, 2010-2020, and after 2020), geographic region (Asia, Europe, and North America), study design (case-control vs. cohort), and sample size (<1,000 vs. ≥1,000). Potential publication bias was evaluated using Egger’s test (20).

## Results

### Study characteristics

**Figure 1:**
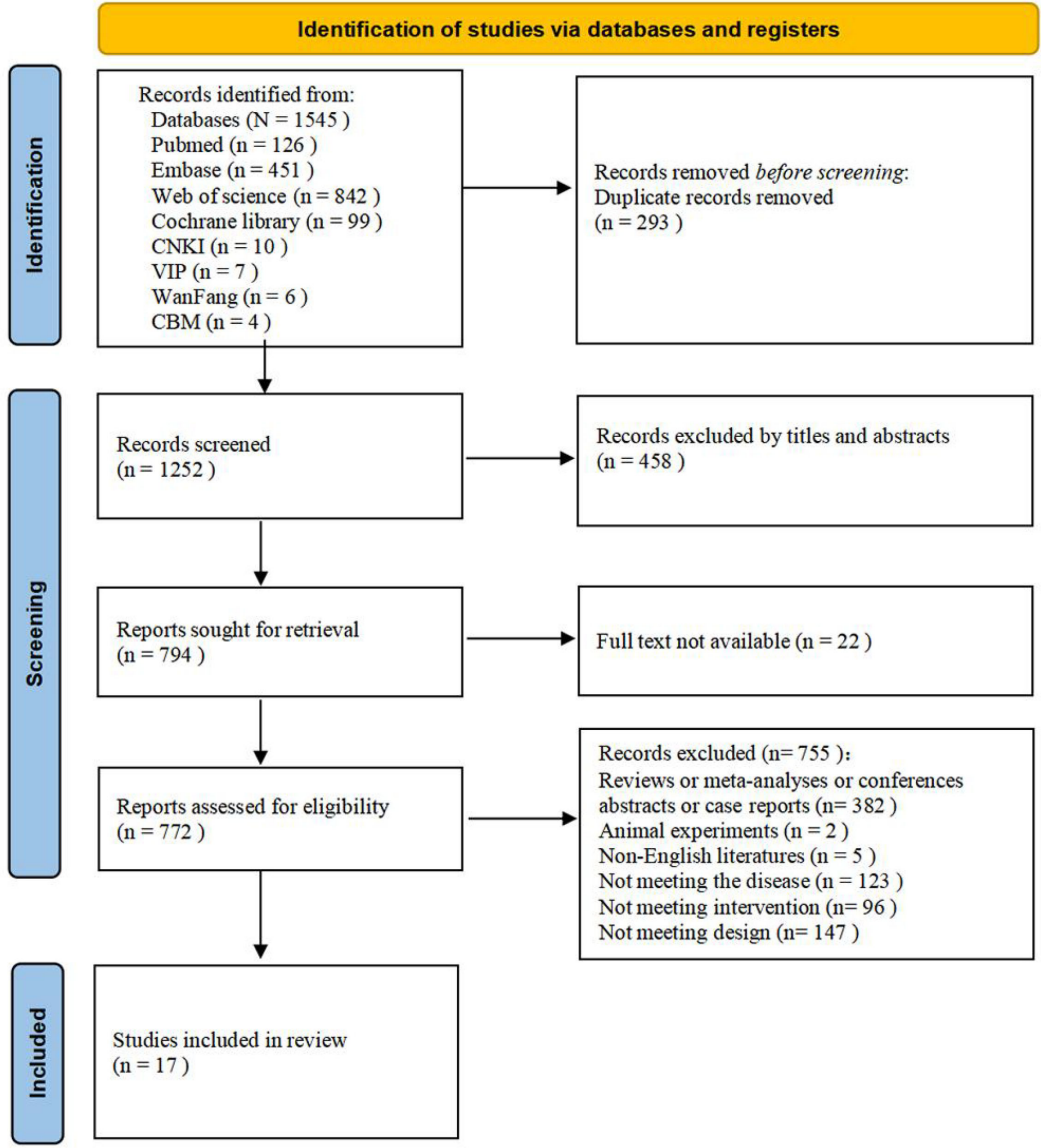
PRISMA 2020 flow diagram of the study selection process for the systematic review and meta-analysis.

EndNote 21 was used to manage references. A total of 293 duplicate records were removed. Based on titles and abstracts, 458 articles were excluded for failing to meet the inclusion criteria. An additional 22 studies were excluded due to unavailability of the full text, and 755 studies, including case reports, reviews, and animal studies, were also excluded. Ultimately, 17 studies (5, 21–36) were deemed eligible for inclusion in the systematic review. The study selection process is illustrated in the PRISMA flow diagram (**Figure 1**).

A total of 17 studies (seven case-control and ten cohort) comprising 472,150 patients were included in this meta-analysis. Although cross-sectional studies were eligible, no studies of this design met the final inclusion criteria. Geographically, 11 studies were conducted in Asia, 4 in North America, and 2 in Europe. The publication years ranged from 2009 to 2024. Additionally, seven specifically involved patients treated with Janus kinase (JAK) inhibitors. Regarding preventive measures, data on HZ vaccination status were available in 4 studies. Detailed characteristics of the studies and patient populations are summarized in **Table 1**.

**Table 1:** Sociodemographic characteristics of studies included in this systematic review and meta-analysis.

Study	Country/ies	RA	HZ Prevalence	Methods for detecting RA/HZ	Regression model	study design	Female/Male with HZ	Use of JAK inhibitors	HZ Vaccination, n	Age (years), mean ± SD	Duration of RA (years/months), mean ± SD	Reported risk factor in multivariable analysis
Wang MR2021 (21)	Asia, China	2577	1.3%	The 2010 ACR/EULAR revised classification of RA standard/Chinese Expert consensus on herpes zoster formulated in 2018	logistic regression	Case-control study	25/9	NR	NR	RA: 57.8 ± 13.2 with HZ: 61.0 ± 10.7 without HZ: NR	with HZ: 8.85 ± 10.14 without HZ: 8.37 ± 8.78	Hypertriglyceridemia, Use multiple DMARDs, Absolute value of lymphocytes
Mo HL2019 (22)	Asia, China	54	18.5%	The 2010 ACR/EULAR revised classification of RA standard/Guidelines issued by the German Society of Dermatology in 2003	logistic regression	Case-control study	8/2	Yes	NR	RA: NR with HZ: 61.90 ± 10.42 without HZ: 58.48 ± 12.96	with HZ: 8.2 ± 2.7* without HZ: 13.7 ± 9.6*	Number of hospitalizations, Use of glucocorticoids/DMARDs within the last 1 month, Age
Chen YJ 2020 (24)	Asia, Taiwan, China	125	5.6%	The 2010 ACR/EULAR revised classification of RA standard/Medical records,clinical assessment, use of antiviral therapy	NR	case-control study	6/1	NR	0	RA: ≥18 with HZ: 56.2 ± 6.2 without HZ: 58.2 ± 3.9	with HZ: 5.5 ± 2.2 without HZ: 11.8 ± 1.2	NR
Cito 2024 (36)	Europe, Italy	198	4.5%	The 2010 ACR/EULAR revised classification of RA standard/Clinical assessment	logistic regression	cohort study	8/1	NR	198	RA: NR with HZ: 58.7 ± 16.7 without HZ: 54.1 ± 13	with HZ: 19.8 ± 5.9* without HZ: 11.5 ± 4.7*	NR
Dlamini 2023 (27)	Asia, Taiwan, China	1651	15.7%	Clinical and laboratory evaluations	logistic regression	cohort study	224/35	NR	NR	RA: 46.2 ± 12.9 with HZ: 50 ± 12.1 without HZ: 45.5 ± 12.9	NR	Use of prednisolone/biopharmaceutical/DMARDs
Harada 2017 (23)	Asia, Japan	257	16.7%	The 1987 ACR revised classification of RA standard/Medical records, use of antiviral therapy	logistic regression	cohort study	37/6	NR	NR	RA: NR with HZ: 64.1 ± 3.4 without HZ: 63.5 ± 2.7	with HZ: 8.1 ± 3 without HZ: 7.1 ± 2.1	Use of prednisolone/TNFi
Liao 2017a (31)	Asia, Taiwan, China	1375	20%	The 1987 ACR revised classification of RA standard/Medical records, use of antiviral therapy	logistic regression	case-control study	215/60	NR	NR	RA: NR with HZ: 55.3 ± 12.7 without HZ: 55.3 ± 12.7	with HZ: 10 ± 4.7 without HZ: 10.1 ± 4.7	Use of anti-rheumatic medication
Liao 2017 (35)	Asia, Taiwan, China	27609	10.47%	The 1987 ACR revised classification of RA standard/Medical records, use of antiviral therapy	cox regression	cohort study	NR	NR	2	RA: ≥18 with HZ: NR without HZ: NR	NR	Use of anti-rheumatic medication, Hospitalization, Mortality, Age, Sex
Mcdonald 2009 (26)	North America, USA	3661	2.6%	RA Diagnosis Code/Medical records, use of antiviral therapy	cox regression	cohort study	10/86	NR	NR	RA: NR with HZ: 58.5 ± 12 without HZ: 57.8 ± 11.7	NR	Age, Male sex, Prednisone use, Hypertension, Diabetes mellitus, Malignancy, Chronic lung disease, Renal failure, Liver disease, AIDS
Pappas 2015 (25)	North America, USA	28852	2.5%	RA Diagnosis Code/Medical records	cox regression	cohort study	NR	NR	NR	RA: 58.14 ± 13.48 with HZ: NR without HZ: NR	NR	Age, Female, mHAQ, Duration RA Prednisone dose, diabetes mellitus, Malignancy, Stroke, CVD, No. prior biologic agents, Current medications
Ryu 2021 (33)	Asia, South Korea	285792	0.65%	RA Diagnosis Code/Medical records, use of antiviral therapy	cox regression	cohort study	1504/365	NR	0	RA: 53.4 ± 15.8 with HZ: 51.2 ± 15.4 without HZ: 53.4 ± 15.8	with HZ: 1.3 ± 1.1 without HZ: 2.4 ± 1.4	Age, Female, Comorbidities, Rheumatic disease, Medications
Sakai 2018 (30)	Asia, Japan	6712	2.9%	RA Diagnosis Code/Medical records, use of antiviral therapy	logistic regression	cohort study	NR	Yes	NR	NR	NR	Previous HZ, Chronic pulmonary Disease, Renal disease, Oral corticosteroid use
Song 2022 (32)	Asia, South Korea	1147	5.3%	The 2010 ACR/EULAR revised classification of RA standard/Clinical assessment	logistic regression	case-control study	55/6	Yes	NR	RA: ≥19 with HZ: 58.1 ± 9.9 without HZ: 55 ± 13.7	with HZ: 9.8 ± 8.3 without HZ: 11.3 ± 8.5	NR
Strangfeld 2009 (34)	Europe, Germany	5040	1.7%	NR	cox regression	cohort study	NR	Yes	NR	NR	NR	Age, Sex, Duration RA, Rheumatoid factor, Medications
Tanaka 2021 (28)	Asia, Japan	101498	2.5%	RA Diagnosis Code/Medical records, use of antiviral therapy	logistic regression	case-control study	1910/656	Yes	NR	RA: ≥18 with HZ: 62.7 ± 13 without HZ: 61.8 ± 14	NR	Hypertension, Dyslipidemia, diabetes mellitus
Veetil 2013 (29)	North America, USA	813	10.3%	The 2010 ACR/EULAR revised classification of RA standard/Clinical assessment	cox regression	cohort study	NR	Yes	NR	RA: 55.9 ± 15.7 with HZ: 51.2 ± 15.4 without HZ: 53.4 ± 15.8	NR	Rheumatoid factor positive, Current smoker, BMI, Joint surgery, Medications
Winthrop 2014 (5)	North America, USA	4789	5.0%	RA Diagnosis Code/Medical records, use of antiviral therapy	logistic regression	case-control study	208/31	Yes	NR	RA: ≥18 with HZ: 56.4 ± 9.7 without HZ: 54 ± 9.3	NR	Age, Duration RA,

NR, Not Reported; RA, Rheumatoid arthritis; HZ, Herpes zoster; DMARDs, Disease Modifying Antirheumatic drugs; *=Duration of RA (months); ACR, American College of Rheumatology; EULAR, European League Against Rheumatism; TNFi, tumor necrosis factor; JAK, Janus kinase; AIDS, acquired immune deficiency syndrome; mHAQ, modified Health Assessment Questionnaire; CVD, cardiovascular disease.

### Quality assessment

The quality assessment of included studies is presented in **Table 2** of the **Supplementary Materials**. All studies scored above 6 points on the Newcastle-Ottawa Scale (NOS), with a mean score of 7.76 (SD = 0.42), indicating that the overall quality of the included studies was high.

### Meta-analysis of HZ pooled proportion

**Figure 2:**
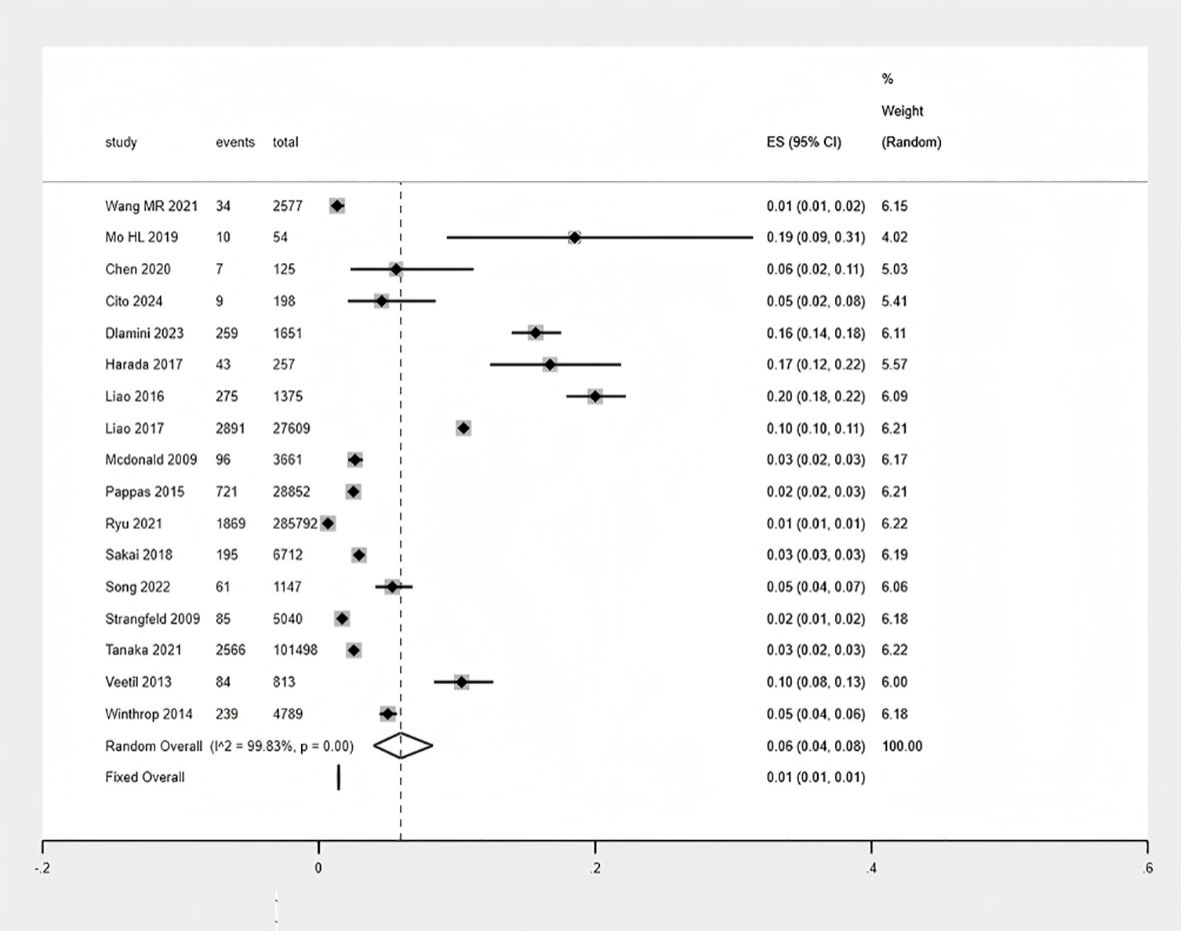
Meta-analysis of the proportion of HZ.

Seventeen studies were included in the quantitative synthesis. The pooled proportion of HZ across the included studies was 6% (95% CI: 4%-8%). However, the 95% prediction interval was calculated as 5% to 45%, indicating that the proportion reported in any future study could vary widely within this range (**Supplementary Figure 1**). Given the high level of heterogeneity among the included studies (I² = 99.83%; P < 0.001), a random-effects model was applied (**Figure 2** Meta-analysis of the proportion of HZ).

### Publication bias

**Figure 3:**
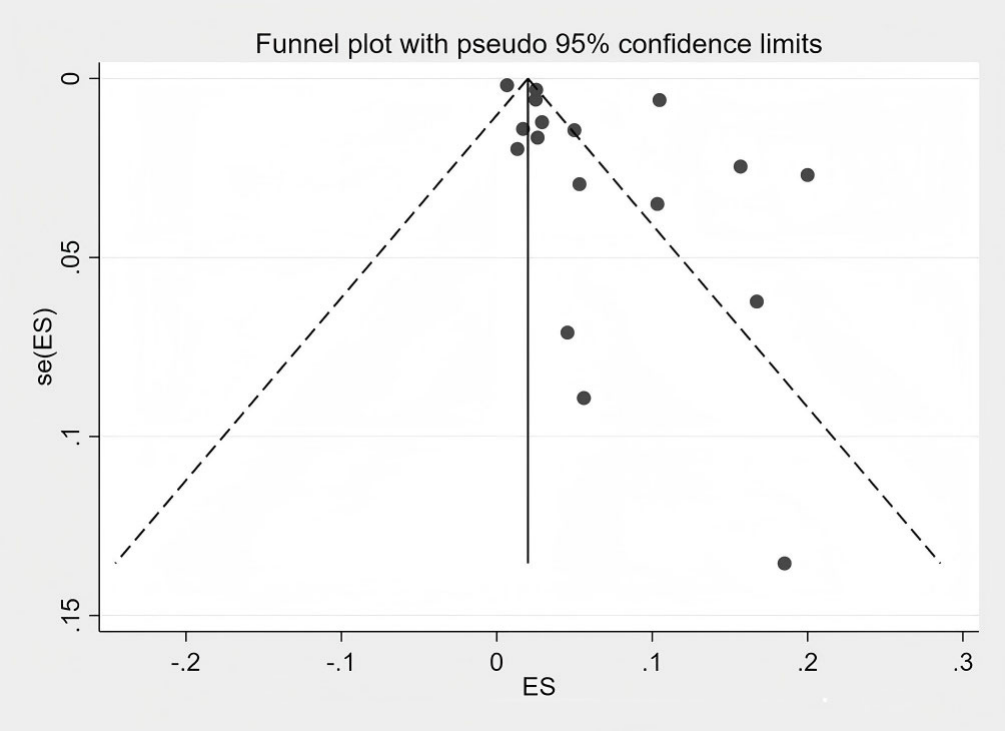
Funnel plot of the proportion of HZ.

**Figure 4:**
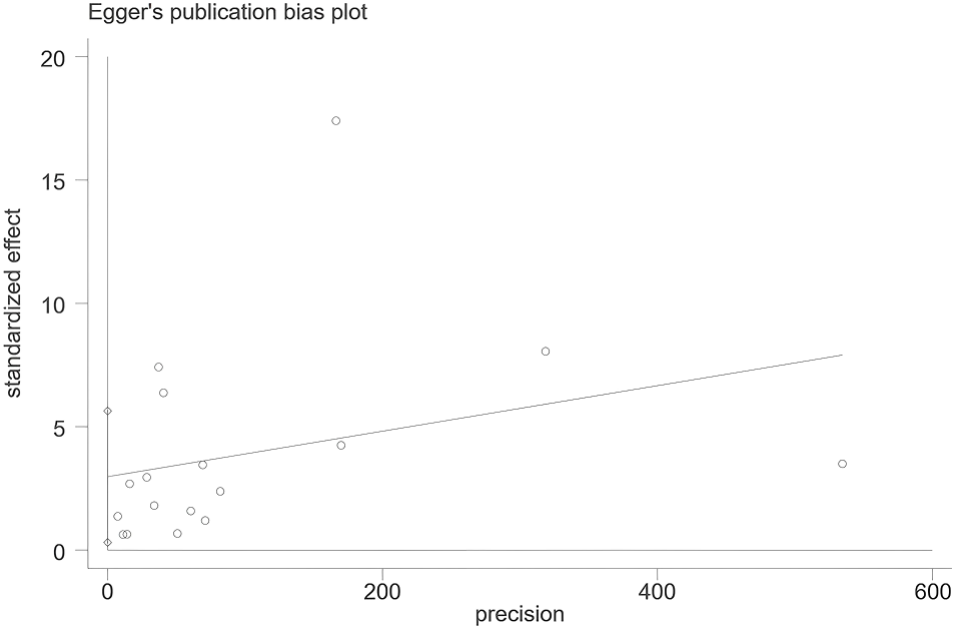
Egger test plot of HZ proportion.

The funnel plot displayed visual asymmetry (**Figure 3**), and Egger’s test yielded a P-value of 0.031, suggesting statistical evidence of asymmetry. However, given the limited number of included studies and the substantial heterogeneity, this asymmetry should be interpreted with caution. It may reflect small-study effects or true heterogeneity in proportion estimates across different populations and settings, rather than solely indicating publication bias. (**Figure 4** Egger test plot of HZ proportion).

### Sensitivity analysis

**Figure 5:**
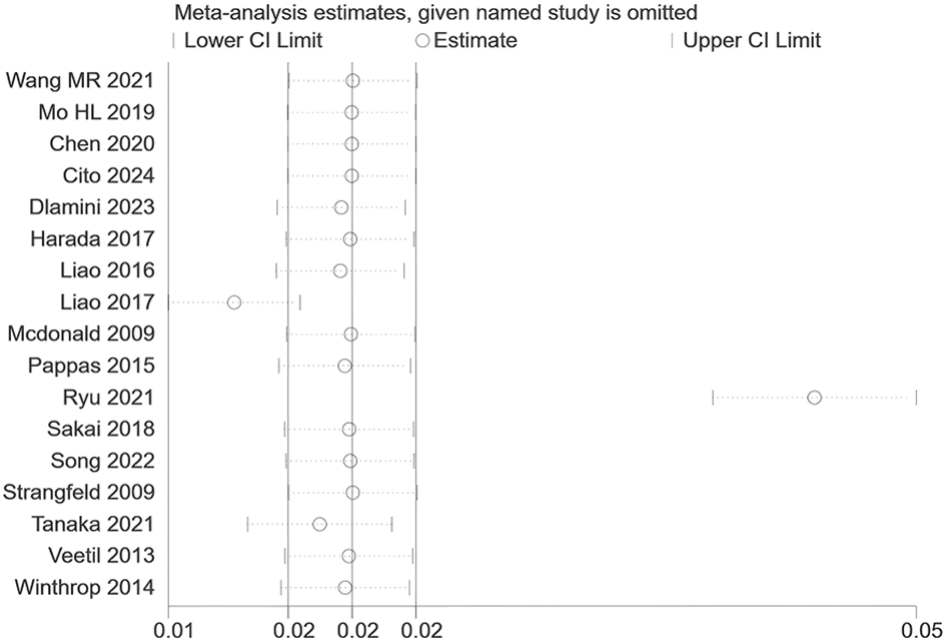
Sensitivity analysis plot of HZ proportion.

Sensitivity analyses using the leave-one-out method confirmed the robustness of our findings. As visualized in **Figure 5**, sequentially omitting individual studies yielded pooled estimates that remained consistent with the primary analysis, demonstrating that the results were not driven by any single study.

### Subgroup analysis

**Figure 6:**
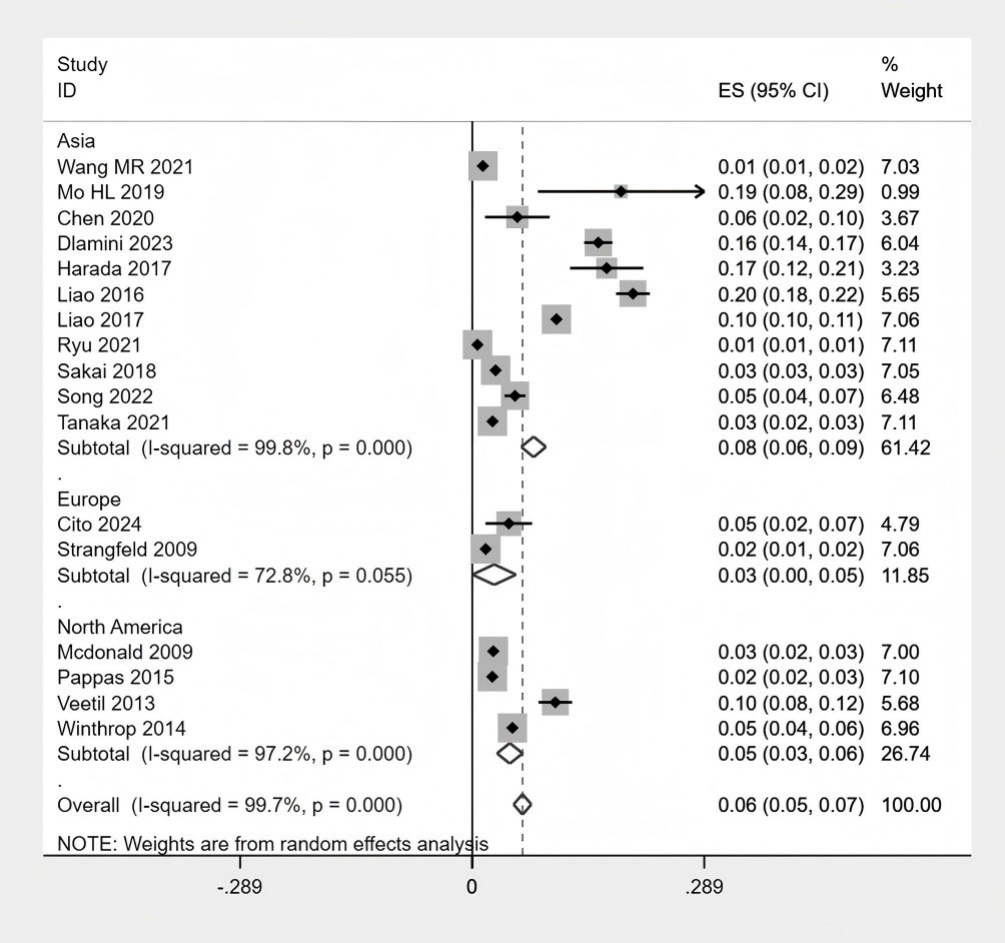
Subgroup analysis of the proportion of HZ in region.

**Figure 7:**
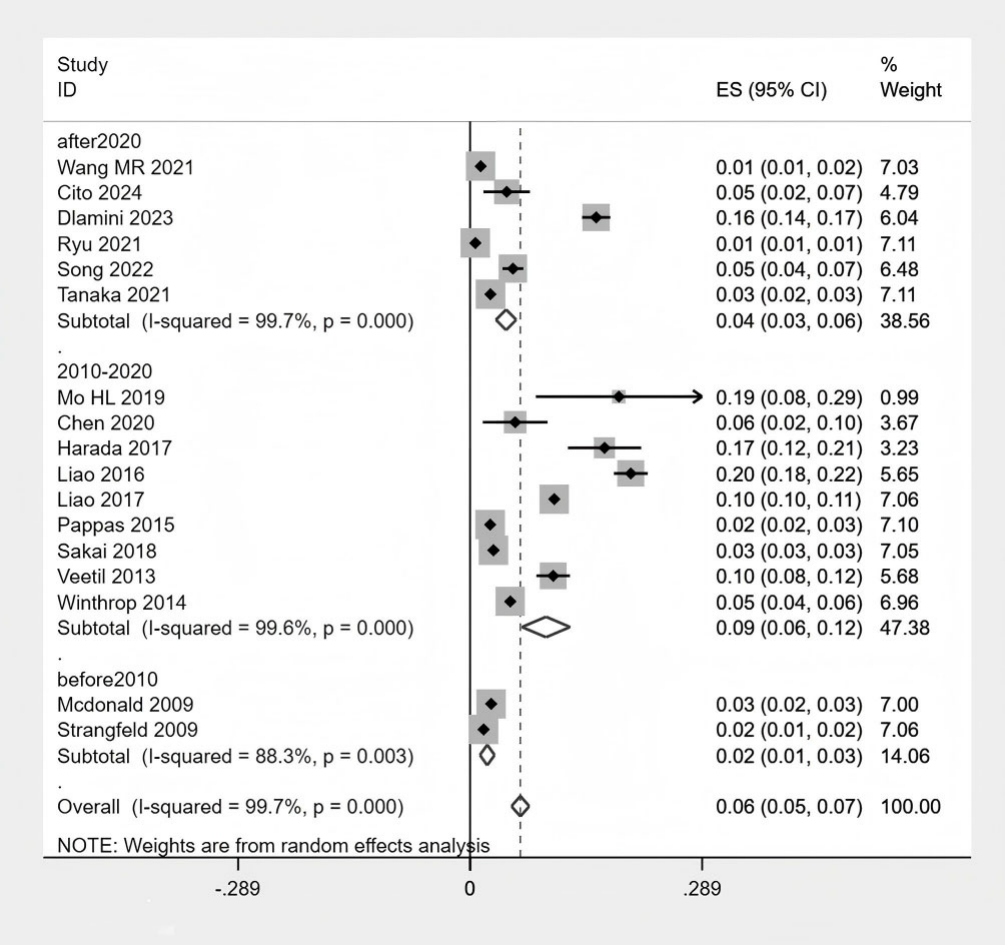
Subgroup analysis of the proportion of HZ in year.

**Figure 8:**
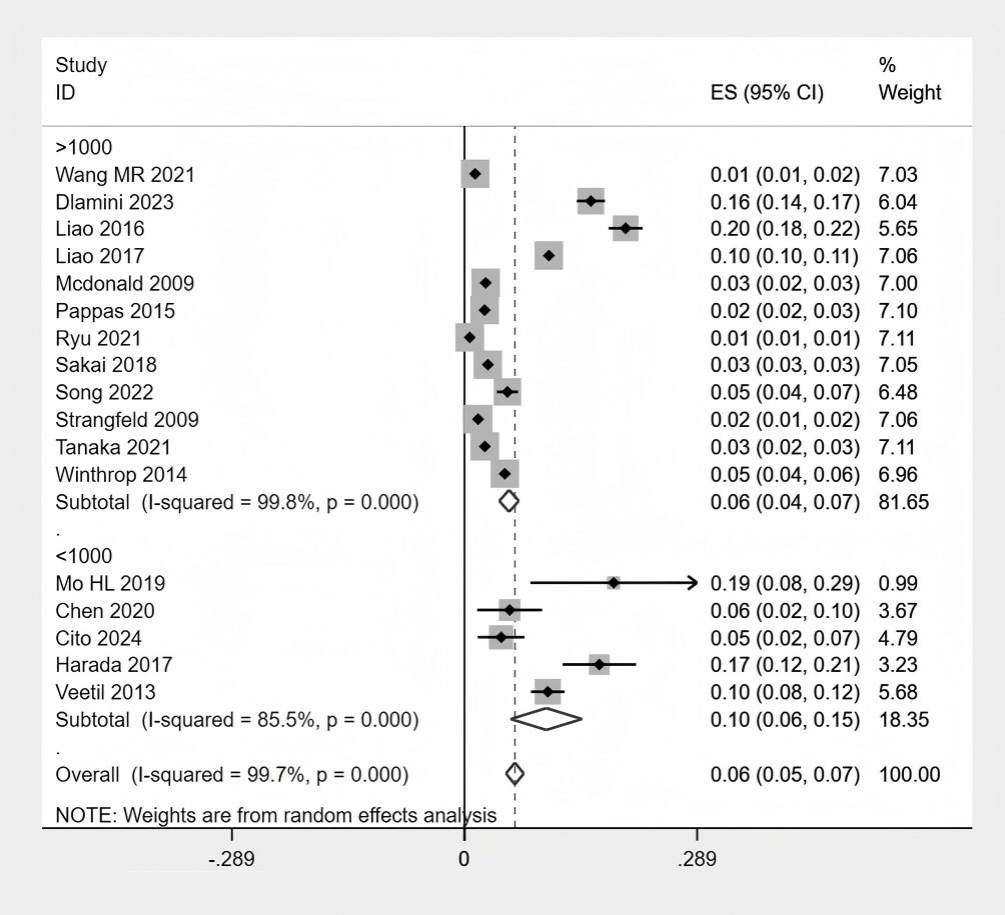
Subgroup analysis of the proportion of HZ in sample size.

**Figure 9:**
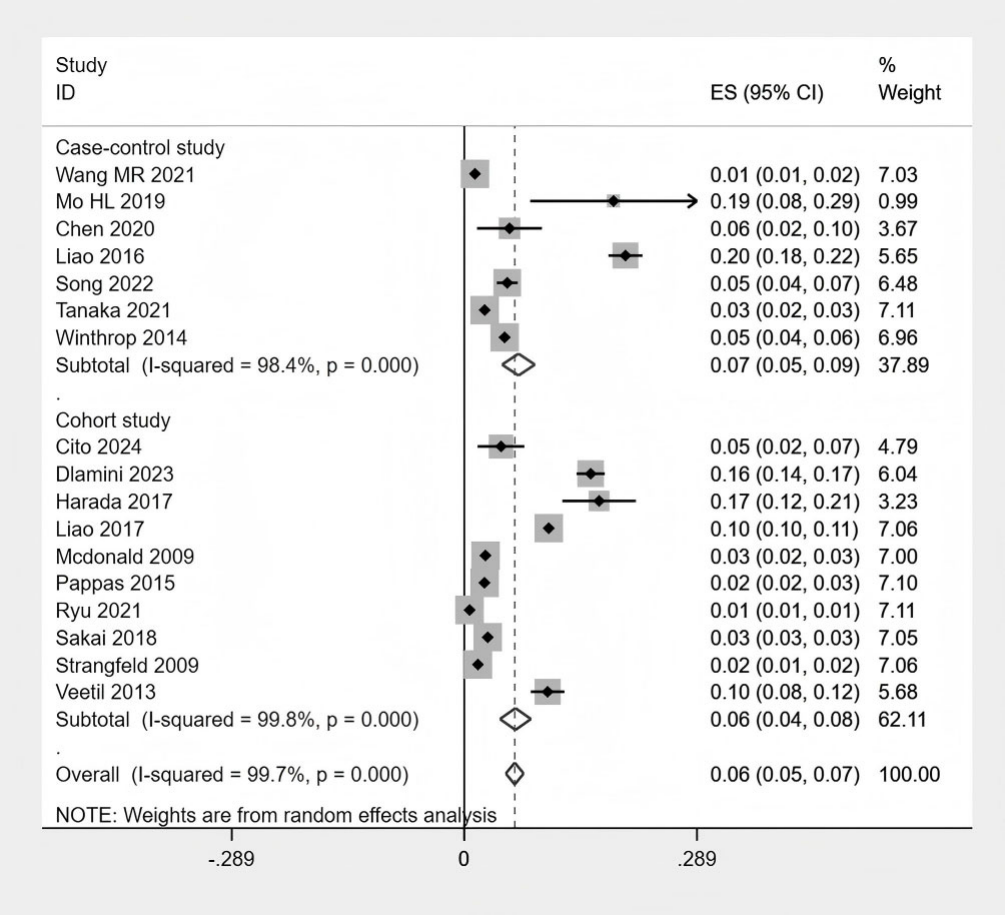
Subgroup analysis of the proportion of HZ in type.

The specific subgroup analysis results can be found in **Table 3** of the **Supplementary Materials**. In exploratory subgroup analyses stratified by geographic region, RA patients in Asia had the highest proportion of HZ (8%), followed by North America (5%) and Europe (3%) (**Figure 6** Subgroup analysis of the proportion of HZ in region).When stratified by publication year, studies published between 2010 and 2020 reported the highest pooled prevalence of HZ (9%), which was significantly higher than studies published before 2010 (4%) and after 2020 (4%) (**Figure 7** Subgroup analysis of the proportion of HZ in year). Regarding study design, there was no significant difference in HZ prevalence between case-control and cohort studies (**Figure 8** Subgroup analysis of the proportion of HZ in sample size). Based on sample size, the prevalence of HZ increased with larger sample sizes (**Figure 9** Subgroup analysis of the proportion of HZ in type). Notably, although these exploratory subgroup analyses based on geographic region, publication year, and sample size resulted in some reduction in I² values, substantial heterogeneity remained. None of these associated factors were identified as major contributors to the observed between-study heterogeneity.

### Results of single factors meta-analysis

#### Female gender

**Figure 10:**
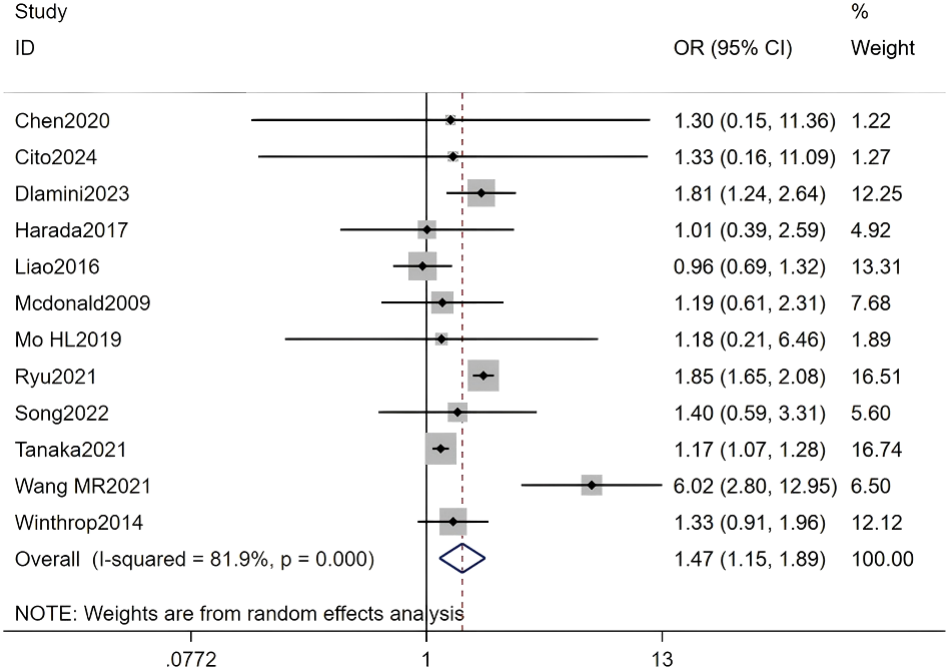
Forest plot of the meta-analysis of female gender.

Twelve studies, including a total of 403,124 RA patients, were analyzed. The meta-analysis indicated that female gender was a significant factor for HZ (OR = 1.47, 95% CI: 1.15-1.89, P = 0.002). Due to substantial heterogeneity among studies (I² = 81.9%, P < 0.001), a random-effects model was applied. Detailed results are presented in **Figure 10** and **Supplementary Material Table 4A** (Forest plot of the meta-analysis for female gender).

#### Use of corticosteroids

**Figure 11:**
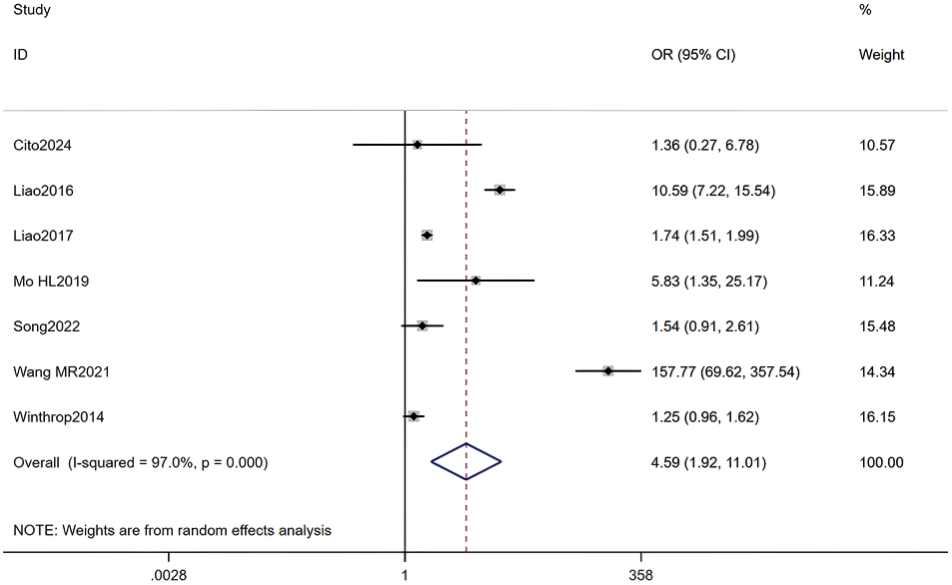
Forest plot of the meta-analysis for corticosteroids use.

Seven studies, including a total of 37,749 RA patients, were analyzed. The meta-analysis indicated that corticosteroid use was a significant associated with HZ (OR = 4.59, 95% CI: 1.92-11.01, P = 0.001). Given the substantial heterogeneity among studies (I² = 97.0%, P < 0.001), a random-effects model was applied. Detailed results are presented in **Figure 11** (Forest plot of the meta-analysis for corticosteroid use).

#### Use of methotrexate

**Figure 12:**
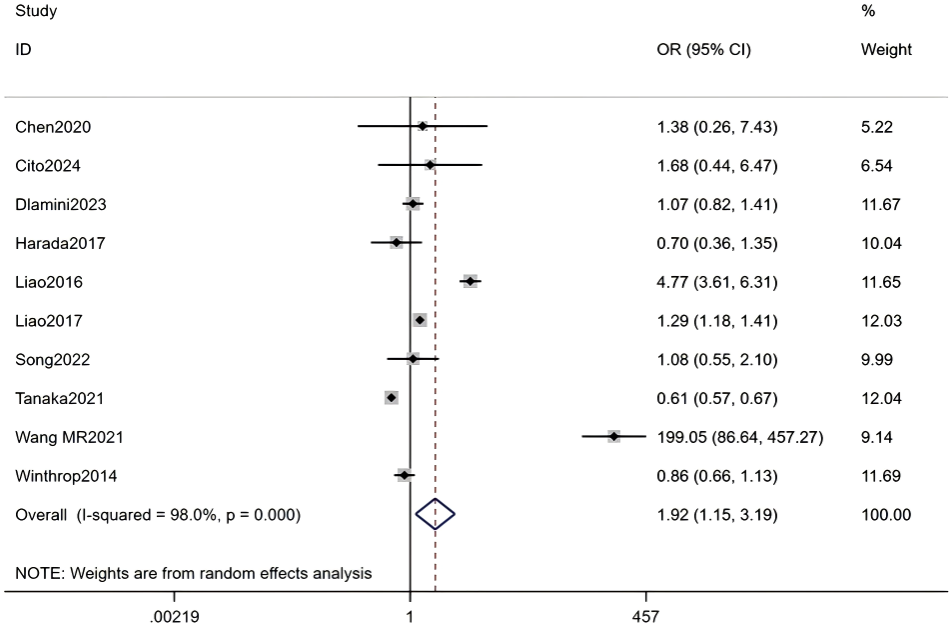
Forest plot of the meta-analysis for methotrexate use.

Ten studies, including a total of 141,226 RA patients, were analyzed. The meta-analysis indicated that methotrexate use was a significant associated with HZ (OR = 1.92, 95% CI: 1.15-3.19, P = 0.012). Due to substantial heterogeneity among studies (I² = 98.0%, P < 0.001), a random-effects model was applied. Detailed results are presented in **Figure 12** (Forest plot of the meta-analysis for methotrexate use).

#### Use of hydroxychloroquine

**Figure 13:**
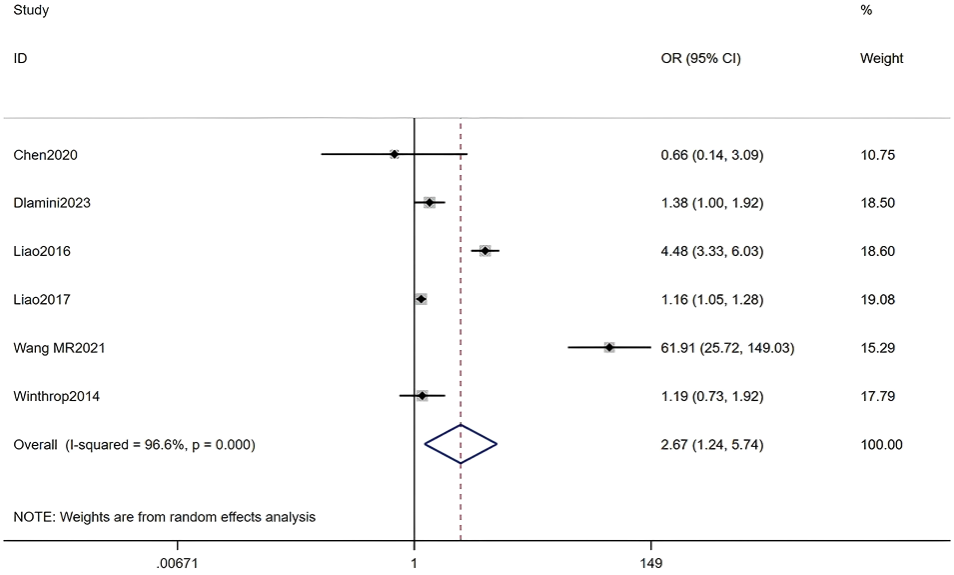
Forest plot of the meta-analysis for hydroxychloroquine use.

Six studies, including a total of 38,126 RA patients, were analyzed. The meta-analysis indicated that hydroxychloroquine use was a significant associated with HZ (OR = 2.67, 95% CI: 1.24-5.74, P = 0.012). Given the substantial heterogeneity among studies (I² = 96.6%, P < 0.001), a random-effects model was applied. Detailed results are presented in **Figure 13** (Forest plot of the meta-analysis for hydroxychloroquine use).

#### History of kidney disease

**Figure 14:**
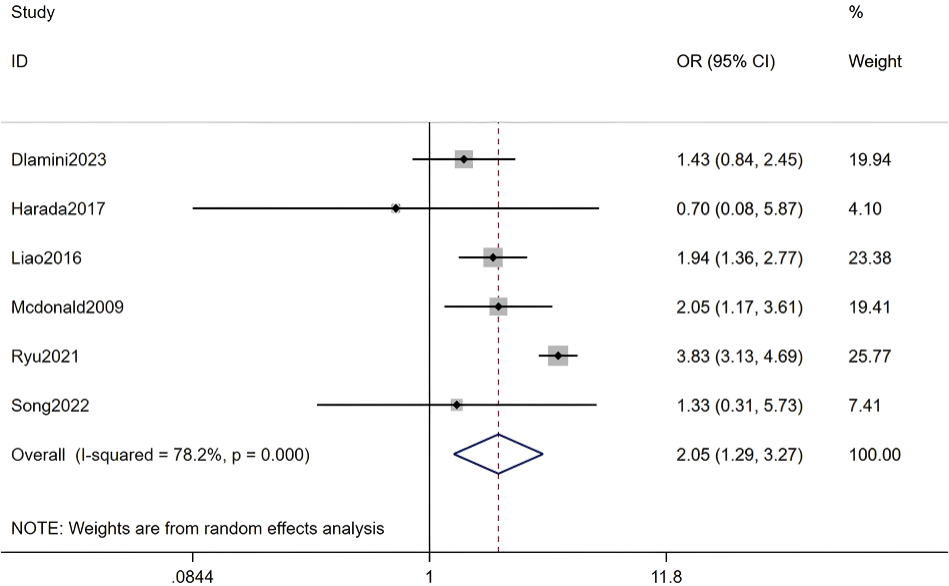
Forest plot of the meta-analysis for history of kidney disease.

Six studies, including a total of 293,883 RA patients, were analyzed. The meta-analysis indicated that a history of kidney disease was a significant factor for HZ (OR = 2.05, 95% CI: 1.29-3.27, P = 0.003). Due to substantial heterogeneity among studies (I² = 78.2%, P < 0.001), a random-effects model was applied. Detailed results are presented in **Figure 14** (Forest plot of the meta-analysis for history of kidney disease).

#### History of hypertension

**Figure 15:**
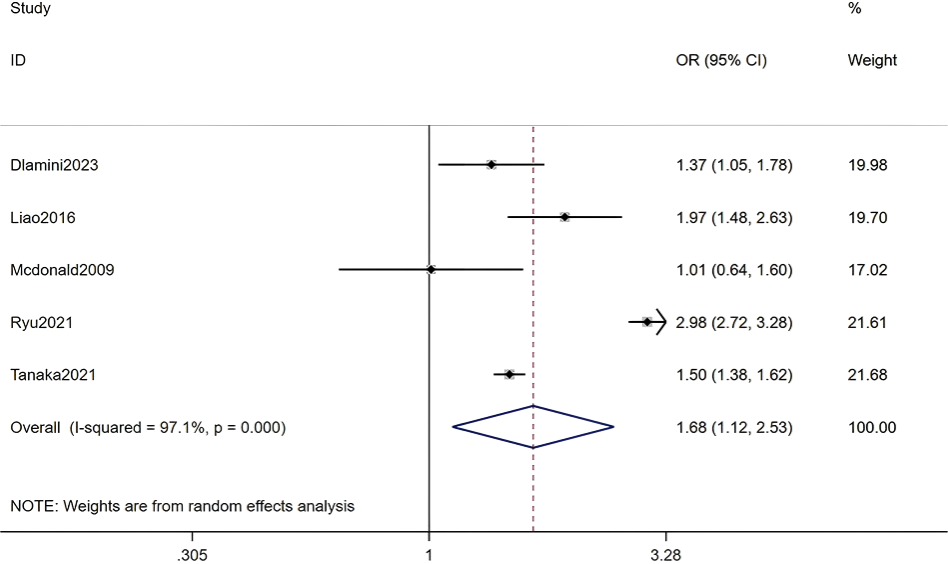
Forest plot of the meta-analysis for history of hypertension.

Five studies, including a total of 393,977 RA patients, were analyzed. The meta-analysis indicated that a history of hypertension was a significant factor for HZ (OR = 1.68, 95% CI: 1.12-2.53, P = 0.012). Given the substantial heterogeneity among studies (I² = 97.1%, P < 0.001), a random-effects model was applied. Detailed results are presented in **Figure 15** (Forest plot of the meta-analysis for history of hypertension).

#### History of heart disease

**Figure 16:**
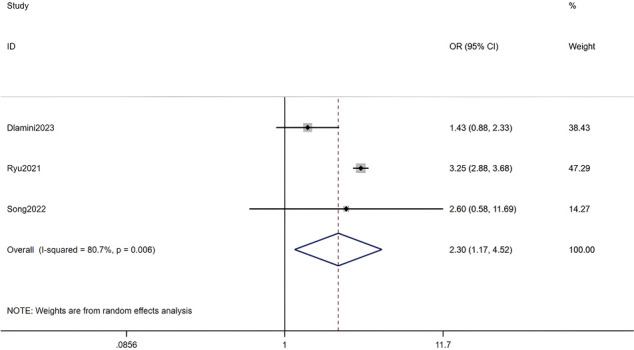
Forest plot of the meta-analysis for history of heart disease.

Three studies, including a total of 288,590 RA patients, were analyzed. The meta-analysis indicated that a history of heart disease was a significant factor for HZ (OR = 2.30, 95% CI: 1.17-4.52, P = 0.016). Given the substantial heterogeneity among studies (I² = 80.7%, P = 0.006), a random-effects model was applied. Detailed results are presented in **Figure 16** (Forest plot of the meta-analysis for history of heart disease).

### Results of multiple factors meta-analysis

The results of multifactorial analyses reported in the included studies were synthesized. Statistically significant associated with HZ included corticosteroid dosage ≥7.5 mg/day (OR = 2.16, 95% CI: 1.85-2.53, P < 0.001), corticosteroid use (OR = 1.42, 95% CI: 1.19-1.69, P < 0.001), use of tumor necrosis factor inhibitors (OR = 1.94, 95% CI: 1.43-2.63, P < 0.001), age (OR = 1.12, 95% CI: 1.02-1.22, P = 0.012), methotrexate use (OR = 1.68, 95% CI: 1.39-2.02, P < 0.001), history of pulmonary disease (OR = 1.42, 95% CI: 1.10-1.83, P = 0.007), history of hypertension (OR = 1.43, 95% CI: 1.15-1.77, P = 0.001), and history of kidney disease (OR = 1.30, 95% CI: 1.04-1.62, P = 0.022) (**Table 4B**, **Supplementary Materials**).

### Other meta-analysis results

No statistically significant associations were observed between HZ risk and age at RA onset, RA duration, 28-joint disease activity score (DAS28), diabetes duration, body mass index (BMI), erythrocyte sedimentation rate (ESR), C-reactive protein (CRP), leflunomide use, sulfasalazine use, anti-citrullinated protein antibody (ACPA) positivity, history of diabetes mellitus, history of cancer, or history of liver disease. For specific details, please refer to **Tables 4A, B** in the **Supplementary Materials**.

## Discussion

This systematic review and meta-analysis, comprising 17 studies with 472,150 participants. Our analysis revealed multiple significant factors associated with HZ susceptibility, including demographic characteristics (female gender, increasing age), pharmacological treatments (corticosteroids, tumor necrosis factor inhibitors, methotrexate, and hydroxychloroquine), and comorbidities (pulmonary disease, hypertension, kidney disease, and heart disease). These findings underscore the complex interplay between the disease burden, treatment regimens, and patient health status.

Our meta-analysis identified a pooled HZ proportion of 6% across the included studies. Crucially, this value represents a descriptive average derived from highly heterogeneous studies rather than a precise estimate of risk for individual patients, and should not be viewed as a generalizable constant. The wide prediction interval of 5% to 45% underscores the substantial variation in HZ burden across different regions, healthcare systems, and patient populations. Consequently, the 6% figure must be interpreted with caution and within the specific context of each study setting. Our findings align with historical trends observed in large administrative databases; for instance, while Smitten et al. (9). reported an incidence of approximately 12/1,000 person-years in the mid-2000s, Singer et al. (2023) utilized recent US data to demonstrate that this figure has risen to 21.5/1,000 person-years in the modern era of widespread targeted therapy (37). This temporal increase is likely a major contributor to the high heterogeneity (I^2^ = 99.8%) observed in our study.

Our findings indicate that female patients with RA are more susceptible to developing HZ. We hypothesize that this susceptibility could be related to fluctuations in estrogen levels. Moreover, since most RA patients are middle-aged or elderly, women in this age group experience significant hormonal changes associated with menopause, which can influence immune function, partly mediated by emotional factors. Ghosh et al. (38) confirmed that menopause-related immune alterations in women may further increase the risk of HZ reactivation.

Notably, the risk of HZ also increases significantly with age. Previous studies have shown that after the age of 50, immune senescence leads to a decline in immune function, resulting in a markedly higher risk of HZ (39). This is consistent with our findings. Specifically, the reduced activity of age-related T cells, which are crucial for controlling VZV, further elevates the risk of infection in the elderly population (40). Indeed, both age and sex are significantly associated with HZ incidence. Therefore, in the prevention and management of RA, particular attention should be given to female and elderly patients. Gender and age differences should be carefully considered when formulating preventive and therapeutic strategies for RA.

We found that the use of glucocorticoids (GC), a type of corticosteroid, and a daily GC dosage exceeding 7.5 mg was associated with an increased probability of HZ in patients with RA. In recent years, GC have often been combined with biological agents for RA treatment (41). Goekoop et al. (42)reported that in patients with newly diagnosed RA, combined use of prednisone at a maintenance dose of 7.5 mg per day demonstrated significant therapeutic effects. However, prolonged GC use can lead to numerous adverse effects, with infection being the most prominent. Previous studies have shown that GC use may result in fungal infections (43) and myocardial infections (44), although consensus on these risks is not unanimous. A meta-analysis published in 2023 (45) indicated that GC might be associated with HZ occurrence in patients with lupus nephritis. Furthermore, a meta-analysis conducted in Germany on GC-related adverse events (46) demonstrated that patients receiving prednisone at doses below 7.5 mg per day experienced fewer adverse events compared with those receiving doses above 7.5 mg per day. We observed a significant association between corticosteroid use and HZ. Notably, the pooled effect estimate differed substantially between the univariate analysis and the multivariable analysis. This discrepancy is likely driven by the adjustment for confounding factors in the multivariable models. The high univariate estimate may reflect confounding by indication, where disease severity drives both steroid use and infection risk. Consequently, the multivariable result provides a more conservative and reliable measure of the independent association.

Tumor necrosis factor inhibitors (TNFi), a type of biological agent for RA treatment, have been widely used in recent years. However, their safety has attracted considerable attention, as TNFi can impair the immune system by blocking tumor necrosis factor. Specifically, they are associated with an increased risk of infectious diseases, including tuberculosis and herpes zoster. Whether TNFi use is associated with HZ in RA patients remains controversial. Previous studies suggested that TNFi represented a high-risk factor for HZ occurrence in RA patients (47), whereas other studies reported no significant correlation between TNFi use and HZ (25, 48). Our findings further support the association between TNFi treatment and an increased risk of HZ in patients with RA. The data suggest that TNFi treatment may be linked to the occurrence of HZ. This might be due to the disease itself or the immunosuppressive treatment, which is hypothesized to lead to an imbalance in the immune system of RA patients, thereby potentially increasing the infection risk of RA patients.

We also found that methotrexate (MTX) is associated with HZ in RA patients. MTX effectively prevents joint damage and is currently the first-line therapy for RA (49); however, evidence regarding its role in increasing HZ risk remains controversial (34, 50). A systematic review (51) reported an elevated risk of HZ infection in RA patients undergoing long-term MTX therapy, whereas McLean et al. (52) suggested that long-term low-dose MTX use does not appear to increase infection risk, which may depend on associated factors such as dosage and treatment duration. We recommend that RA patients receiving MTX closely monitor changes in skin color and morphology in the early stages of treatment, and seek medical attention promptly if HZ develops.

Hydroxychloroquine (HCQ), a derivative of the antimalarial agent chloroquine (53), can inhibit multiple pro-inflammatory cytokines and is used in RA management (54). It has also been reported to alleviate skin symptoms associated with diabetes mellitus (DM) (55). Nevertheless, our findings suggest that HCQ may be associated with HZ in RA patients. HCQ use can result in adverse dermatological effects, including rashes or drug eruptions (56, 57). Therefore, the use of HCQ in RA patients with HZ should be carefully considered based on individual patient circumstances.

We identified that a history of lung disease, hypertension, kidney disease, and heart disease are associated factors for the co-occurrence of HZ in patients with RA. Although these associated factors may appear unrelated, they collectively highlight a central mechanism involving immunosenescence and comorbidity burden (58, 59). Patients with a history of lung or heart disease often experience indirect effects on immune cell metabolism due to associated factors such as tissue hypoxia and malnutrition (60), thereby reducing the body’s antiviral defenses. In patients with advanced chronic kidney disease, lymphocytes and dendritic cells are impaired, leading to immune deficiencies (61) and a markedly increased risk of infection. The elevated inflammatory levels generated by these conditions, combined with the inherent inflammation in RA patients, further disrupt immune homeostasis and weaken immune surveillance against VZV. Although hypertension is traditionally not classified as an inflammatory disease, studies have shown that it is associated with vascular inflammation and immune system dysregulation (62). Persistent high blood pressure causes endothelial dysfunction and oxidative stress (63), creating an unfavorable microenvironment for immune cell survival. Collectively, it is hypothesized that these comorbidities may contribute to accelerated immunosenescence, potentially placing patients at a higher risk of HZ than those without such conditions. Therefore, when assessing HZ risk in RA patients, a holistic approach should be adopted, considering the patient’s overall health rather than focusing solely on the rheumatic disease or its treatment.

To mitigate the substantial HZ burden in RA patients, targeted vaccination strategies are of paramount importance. The impact of active vaccine campaigns in frail and high-risk populations cannot be overstated. Recent real-world studies have consistently demonstrated that the recombinant zoster vaccine (RZV) offers effective medium- to long-term protection against HZ and postherpetic neuralgia in rheumatologic patients, exhibiting a highly favorable safety profile without increasing the risk of severe disease flares (64, 65). Furthermore, broader evidence on immunization in rheumatology confirms that frail patients on biological therapies, such as TNF inhibitors, can safely achieve adequate immunogenicity from vaccines (66). Consequently, incorporating tailored anti-zoster vaccination campaigns into the routine clinical management of RA patients is a critical step in reducing infectious comorbidities.

Key strengths of this study include strict adherence to PRISMA guidelines and a comprehensive search across eight databases, enabling the first systematic review and meta-analysis of HZ epidemiology in RA patients. Nevertheless, several limitations should be acknowledged. First, significant heterogeneity was observed in the pooled proportion of HZ, which remained unexplained despite exploratory subgroup analyses. Second, the comparability of the pooled odds ratios for risk factors is limited by the heterogeneous adjustment strategies employed across the primary studies. Since different studies adjusted for varying sets of confounders, the pooled estimates should be interpreted as observational associations rather than definitive causal effects. Third, only studies published in English and Chinese were included, and unpublished studies were not reviewed, potentially introducing selection bias. Fourth, our findings may not fully represent global burden due to the lack of data from regions such as Africa and South America. Fifth, limited information in the original studies constrained our ability to explore certain risk factors comprehensively. Sixth, the relatively small number of included studies precluded meta-analyses for many specific risk factors. Seventh, there is a notable lack of data regarding anti-zoster vaccination status across the included observational studies. This absence of vaccination records prevented us from adjusting for vaccine-induced protection or evaluating the modifying impact of vaccination strategies in our meta-analysis. Therefore, future large-scale, multicenter studies are warranted to address these limitations and enhance the generalizability of the findings.

## Conclusion

This systematic review and meta-analysis synthesize the available evidence on the pooled proportion of HZ and evaluates factors associated with the infection in RA patients. Our findings indicate that multiple factors are significantly associated with HZ infection, including female gender, age, corticosteroid use and dosage ≥7.5 mg/day, tumor necrosis factor inhibitors, methotrexate, hydroxychloroquine, and a history of pulmonary disease, hypertension, kidney disease, and heart disease. Future research should aim to elucidate the precise biological mechanisms underlying these associations and to develop targeted preventive strategies to mitigate HZ risk in this patient population.

## Data Availability

The original contributions presented in the study are included in the article/**Supplementary Material**. Further inquiries can be directed to the corresponding authors.
