# Supplementary material for: Prevalence and risk factors of herpes zoster in patients with rheumatoid arthritis: a systematic review and meta-analysis
Source: Front Immunol. 2026 May 8;17:1754915. doi: 10.3389/fimmu.2026.1754915 (PMC13194116; doi:10.3389/fimmu.2026.1754915)
Supplement: Supplementary file 1 [file DataSheet1.zip › Supplementary Materials/Supplementary Figure 1.docx]

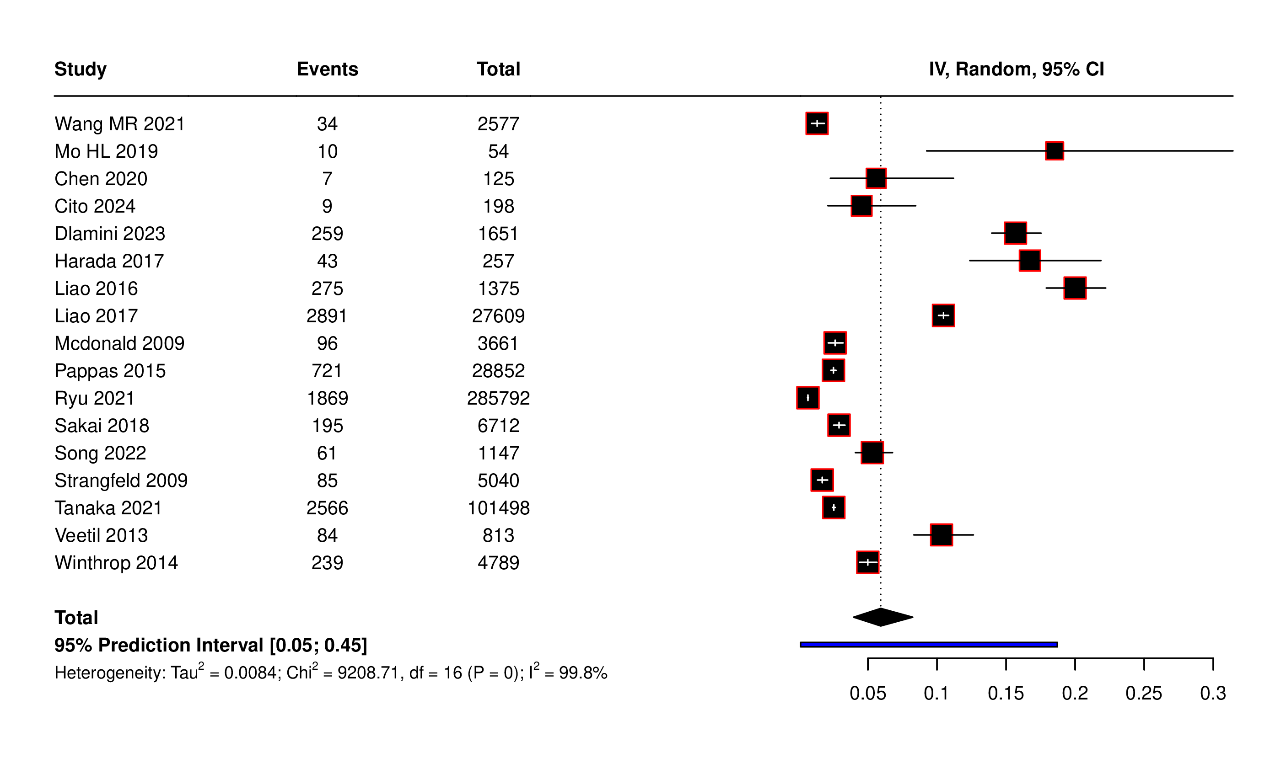


Supplementary figure 1 Forest plot of the descriptive pooled proportion of HZ in patients with rheumatoid arthritis. The horizontal line indicates the 95% prediction interval (5%–45%). Given the substantial between-study heterogeneity, this wide interval illustrates the expected range of HZ proportions in future individual studies or diverse clinical settings, rather than a single universal population risk.
