# Supplementary material for: Prevalence and risk factors of herpes zoster in patients with rheumatoid arthritis: a systematic review and meta-analysis
Source: Front Immunol. 2026 May 8;17:1754915. doi: 10.3389/fimmu.2026.1754915 (PMC13194116; doi:10.3389/fimmu.2026.1754915)
Supplement: Supplementary file 1 [file DataSheet1.zip › Supplementary Materials/Table 1. Search strategy.docx]

**Table 1.** Search strategy

**Table 1-a.** PubMed

| **Items** | **Keywords** | **Results** |
| --- | --- | --- |
| #1 | "Arthritis, Rheumatoid"[Mesh] | 132,295 |
| #2 | (((Rheumatoid Arthritis[Title/Abstract]) OR (Musculoskeletal Diseases[Title/Abstract])) OR (Joint Diseases[Title/Abstract])) OR (Arthritis[Title/Abstract]) | 234,474 |
| #3 | #1 OR #2 | 271,444 |
| #4 | "Herpes Zoster"[Mesh] | 13,919 |
| #5 | (((Herpes Zoster[Title/Abstract]) OR (Shingles[Title/Abstract])) OR (Zona[Title/Abstract])) OR (Zoster[Title/Abstract]) | 37,639 |
| #6 | #4 OR #5 | 40,277 |
| #7 | "Risk Factors"[Mesh] | 1,037,430 |
| #8 | ((((((Risk Factor[Title/Abstract]) OR (Risk Factor*[Title/Abstract])) OR (Population* at Risk[Title/Abstract])) OR (Risk Score*[Title/Abstract])) OR (Risk Factor Score*[Title/Abstract])) OR (Health Correlates[Title/Abstract])) OR (Social Risk Factor*[Title/Abstract]) | 925,125 |
| #9 | #7 OR #8 | 1,554,757 |
| #10 | #3 AND #6 AND #9 | 126 |

**Table 1-b.** Web of Science

| **Items** | **Keywords** | **Results** |
| --- | --- | --- |
| #1 | TS=(Rheumatoid Arthritis) OR TS=(Musculoskeletal Diseases) OR TS=(Joint Diseases) OR TS=(Arthritis) | 1472543 |
| #2 | TS=(Herpes Zoster) OR TS=(Shingles) OR TS=(Zona) OR TS=(Zoster) | 99641 |
| #3 | TS=(Risk Factor*) OR TS=(Population* at Risk) OR TS=(Risk Score*) OR TS=(Risk Factor Score*) OR TS=(Health Correlates) OR TS=(Social Risk Factor*) | 4185450 |
| #4 | #1 AND #2 AND #3 | 842 |

**Table 1-c.** Embase

| **Items** | **Keywords** | **Results** |
| --- | --- | --- |
| #1 | 'rheumatoid arthritis'/exp | 279417 |
| #2 | 'rheumatoid arthritis':ab,ti | 202271 |
| #3 | 'musculoskeletal diseases':ab,ti | 4004 |
| #4 | 'joint diseases':ab,ti | 5039 |
| #5 | 'arthritis':ab,ti | 338070 |
| #6 | #1 OR #2 OR #3 OR #4 OR #5 | 420081 |
| #7 | 'herpes zoster'/exp | 36134 |
| #8 | 'herpes zoster':ab,ti | 17527 |
| #9 | 'shingles':ab,ti | 2781 |
| #10 | 'zona':ab,ti | 17255 |
| #11 | 'zoster':ab,ti | 30802 |
| #12 | #7 OR #8 OR #9 OR #10 OR #11 | 63909 |
| #13 | 'risk factor'/exp | 1556509 |
| #14 | 'risk factor':ab,ti | 445204 |
| #15 | 'risk factor*':ab,ti | 1279920 |
| #16 | 'population* at risk':ab,ti | 10356 |
| #17 | 'risk score*':ab,ti | 74803 |
| #18 | 'risk factor score*':ab,ti | 444 |
| #19 | 'health correlates':ab,ti | 690 |
| #20 | 'social risk factor*':ab,ti | 1669 |
| #21 | #13 OR #14 OR #15 OR #16 OR #17 OR #18 OR #19 OR #20 | 2046772 |
| #22 | #6 AND #12 AND #21 | 451 |

**Table 1-d.** Cochrane Library

| **Items** | **Keywords** | **Results** |
| --- | --- | --- |
| #1 | MeSH descriptor: [Arthritis, Rheumatoid] explode all trees | 8115 |
| #2 | (Rheumatoid Arthritis):ti,ab,kw OR (Musculoskeletal Diseases):ti,ab,kw OR (Joint Diseases):ti,ab,kw OR (Arthritis):ti,ab,kw | 37111 |
| #3 | #1 or #2 | 37517 |
| #4 | MeSH descriptor: [Herpes Zoster] explode all trees | 798 |
| #5 | (Herpes Zoster):ti,ab,kw OR (Shingles):ti,ab,kw OR (Zona):ti,ab,kw OR (Zoster):ti,ab,kw | 3505 |
| #6 | #4 or #5 | 3505 |
| #7 | MeSH descriptor: [Risk Factors] explode all trees | 37757 |
| #8 | (Risk Factor*):ti,ab,kw OR (Population* at Risk):ti,ab,kw OR (Risk Score*):ti,ab,kw OR (Risk Factor Score*):ti,ab,kw OR (Health Correlates):ti,ab,kw | 191415 |
| #9 | (Social Risk Factor*):ti,ab,kw | 7630 |
| #10 | #7 or #8 or #9 | 191415 |
| #11 | #3 and #6 and #10 | 99 |

**Table 1-e.** Search strategies for Chinese Database

| **Database** | **Keywords** |
| --- | --- |
| China Knowledge Resource Integrated Database (CNKI) | 检索式：SU=('类风湿性关节炎患者'+'类风湿性关节炎'+'类风湿关节炎'+'类风关'+'类风湿性病') AND SU=('带状疱疹患者'+'带状疱疹'+'带状疮疹'+'带状性疱疹'+'蜘蛛疮'+'蛇串疮') AND SU=('患病率'+'发生率'+'流行率'+'危险因素'+'影响因素'+'相关因素')  Search Terms: SU=('leifengshixingguanjieyanhuanzhe'+'leifengshixingguanjieyan'+'leifengshiguanjieyan'+'leifengguan'+'leifengshixingbing') AND SU=('daizhuangpaozhenhuanzhe'+ 'daizhuangpaozhen'+ 'daizhuangchuangzhen'+'daizhuanxingpaozhen' +'shechuanchuang' ) AND SU=('huanbinglv'+'fashenglv'+'liuxinglv'+'weixianyinsu'+'yingxiangyinsu'+'xiangguanyinsu') |
| Weipu Database (VIP) | 检索式：M=(类风湿性关节炎患者 OR 类风湿性关节炎 OR 类风湿关节炎 OR 类风关 OR 类风湿性病) AND M=(带状疱疹患者 OR 带状疱疹 OR 带状疮疹 OR 带状性疱疹 OR 蜘蛛疮 OR 蛇串疮) AND M=(患病率 OR 发生率 OR 流行率 OR 现况调查 OR 危险因素 OR 影响因素 OR 相关因素)  Search Terms: M=(leifengshixingguanjieyanhuanzhe OR leifengshixingguanjieyan OR leifengshiguanjieyan OR leifengguan OR leifengshixingbing) AND M=(daizhuangpaozhenhuanzhe OR daizhuangpaozhen OR daizhuangchuangzhen OR daizhuanxingpaozhen OR shechuanchuang) AND M=(huanbinglv OR fashenglv OR liuxinglv OR xiankuangdiaocha OR weixianyinsu OR yingxiangyinsu OR xiangguanyinsu) |
| Wanfang Database | 检索式：主题:(类风湿性关节炎患者 OR 类风湿性关节炎 OR 类风湿关节炎 OR 类风关 OR 类风湿性病) and 主题:(带状疱疹患者 OR 带状疱疹 OR 带状疮疹 OR 带状性疱疹 OR 蜘蛛疮 OR 蛇串疮) and ((主题:(患病率 OR 发生率 OR 流行率 OR 负担 OR 现况调查 OR 现况研究) OR 主题:(危险因素 OR 影响因素 OR 相关因素))  Search Terms: Subject:(leifengshixingguanjieyanhuanzhe OR leifengshixingguanjieyan OR leifengshiguanjieyan OR leifengguan OR leifengshixingbing) and Subject:(daizhuangpaozhenhuanzhe OR daizhuangpaozhen OR daizhuangchuangzhen OR daizhuanxingpaozhen OR shechuanchuang) and ((Subject:(huanbinglv OR fashenglv OR liuxinglv OR fudan OR xiankuangdiaocha OR xiankuangyanjiu) OR Subject:(weixianyinsu OR yingxiangyinsu OR xiangguanyinsu)) |
| China Biology Medicine Database （CBM) | 检索式： ("类风湿性关节炎患者" OR "类风湿性关节炎" OR "类风湿关节炎" OR "类风关" OR "类风湿性病") AND ("带状疱疹患者" OR "带状疱疹" OR "带状疮疹" OR "带状性疱疹" OR "蜘蛛疮" OR "蛇串疮") AND ("患病率" OR "发生率" OR "流行率" OR "危险因素" OR "影响因素" OR "相关因素")  Search Terms: ("leifengshixingguanjieyanhuanzhe" OR "leifengshixingguanjieyan" OR "leifengshiguanjieyan" OR "leifengguan" OR "leifengshixingbing") AND ("daizhuangpaozhenhuanzhe" OR "daizhuangpaozhen" OR "daizhuangchuangzhen" OR "daizhuanxingpaozhen" OR "shechuanchuang") AND ("huanbinglv" OR "fashenglv" OR "liuxinglv" OR "weixianyinsu" OR "yingxiangyinsu" OR "xiangguanyinsu") |
