# Supplementary material for: Prevalence and risk factors of herpes zoster in patients with rheumatoid arthritis: a systematic review and meta-analysis
Source: Front Immunol. 2026 May 8;17:1754915. doi: 10.3389/fimmu.2026.1754915 (PMC13194116; doi:10.3389/fimmu.2026.1754915)
Supplement: Supplementary file 1 [file DataSheet1.zip › Supplementary Materials/Table 2. Quality assessment of included studies.docx]

**Table 2.** Quality assessment of included studies

| **Case control** | | | | | | | | | | |
| --- | --- | --- | --- | --- | --- | --- | --- | --- | --- | --- |
| **No.** | **Study** | **Is the case definition adequate?** | **Representativeness of the cases** | **Determination of control group** | **Definition of Controls** | **Comparability of cases and controls based on the design or analysis** | **Ascertainment of exposure** | **Same method of ascertainment for cases and controls** | **Non response** | **Total** |
| 1 | WangMR2021 | 1 | 1 | 1 | 1 | 1 | 1 | 1 | 1 | 8 |
| 2 | Mo HL2019 | 1 | 1 | 1 | 1 | 1 | 1 | 1 | 1 | 8 |
| 3 | Chen2020 | 1 | 0 | 1 | 1 | 1 | 1 | 1 | 1 | 7 |
| 4 | Liao2016 | 1 | 1 | 1 | 1 | 1 | 1 | 1 | 1 | 8 |
| 5 | Song2022 | 1 | 1 | 1 | 1 | 1 | 1 | 1 | 1 | 8 |
| 6 | Tanaka2021 | 1 | 1 | 1 | 1 | 1 | 1 | 1 | 1 | 8 |
| 7 | Winthrop2014 | 1 | 1 | 1 | 1 | 1 | 1 | 1 | 1 | 8 |

**Table 2.** Continued

| **Cohort study** | | | | | | | | | | |
| --- | --- | --- | --- | --- | --- | --- | --- | --- | --- | --- |
| **No.** | **Study** | **Representativeness of the exposed group** | **Selection of non-exposed groups** | **Determination of exposure factors** | **Identification of outcome indicators not yet to be observed at study entry** | **Comparability of exposed and unexposed groups considered in design and statistical analysis** | **Design and statistical analysis** | **Adequacy of the study's evaluation of the outcome** | **Adequacy of follow-up in exposed and unexposed groups** | **Total** |
| 8 | Cito2024 | 1 | 1 | 1 | 1 | 0 | 1 | 1 | 1 | 7 |
| 9 | Dlamini2023 | 1 | 1 | 1 | 0 | 1 | 1 | 1 | 1 | 7 |
| 10 | Harada2017 | 1 | 1 | 1 | 1 | 1 | 1 | 1 | 1 | 8 |
| 11 | Liao2017 | 1 | 1 | 1 | 1 | 1 | 1 | 1 | 1 | 8 |
| 12 | Mcdonald2009 | 1 | 1 | 1 | 1 | 1 | 1 | 1 | 1 | 8 |
| 13 | Pappas2015 | 1 | 0 | 1 | 1 | 1 | 1 | 1 | 1 | 7 |
| 14 | Ryu2021 | 1 | 1 | 1 | 1 | 1 | 1 | 1 | 1 | 8 |
| 15 | Sakai2018 | 1 | 1 | 1 | 1 | 1 | 1 | 1 | 1 | 8 |
| 16 | Strangfeld2009 | 1 | 1 | 1 | 1 | 1 | 1 | 1 | 1 | 8 |
| 17 | Veetil2013 | 1 | 1 | 1 | 1 | 1 | 1 | 1 | 1 | 8 |
