# Supplementary material for: Prevalence and risk factors of herpes zoster in patients with rheumatoid arthritis: a systematic review and meta-analysis
Source: Front Immunol. 2026 May 8;17:1754915. doi: 10.3389/fimmu.2026.1754915 (PMC13194116; doi:10.3389/fimmu.2026.1754915)
Supplement: Supplementary file 1 [file DataSheet1.zip › Supplementary Materials/Table 3. Results of subgroup analysis.docx]

**Table 3.** Subgroup analysis of the prevalence of HZ in patients with RA

| **Subgroup analysis** | **No. of studies** | **Results of heterogeneity test** | | **Results of Meta-analysis** | |
| --- | --- | --- | --- | --- | --- |
|  |  | ***I*^2^ (%)** | ***P* value** | **Effect, 95%*CI*** | ***P* for interaction** |
| All | 17 | 99.7 | 0.000 | 0.06（0.05, 0.07） | P<0.001 |
| year |  |  |  |  | P<0.001 |
| before 2010 | 2 | 88.3 | 0.003 | 0.02（0.01, 0.03） |  |
| 2010-2020 | 9 | 99.6 | 0.000 | 0.09（0.06, 0.12） |  |
| after2020 | 6 | 99.7 | 0.000 | 0.04（0.03, 0.06） |  |
| region |  |  |  |  | P<0.001 |
| Asia | 11 | 99.8 | 0.000 | 0.08（0.06, 0.09） |  |
| Europe | 2 | 72.8 | 0.055 | 0.03（0.00, 0.05） |  |
| North America | 4 | 97.2 | 0.000 | 0.05（0.03, 0.06） |  |
| design |  |  |  |  | P<0.001 |
| Case-control study | 7 | 98.4 | 0.000 | 0.07（0.05, 0.09） |  |
| Cohort study | 10 | 99.8 | 0.000 | 0.06（0.04, 0.08） |  |
| Sample size |  |  |  |  | P<0.001 |
| <1000 | 5 | 85.5 | 0.000 | 0.10（0.06, 0.15） |  |
| >1000 | 12 | 99.8 | 0.000 | 0.06（0.04, 0.07） |  |
