# Supplementary material for: Prevalence and risk factors of herpes zoster in patients with rheumatoid arthritis: a systematic review and meta-analysis
Source: Front Immunol. 2026 May 8;17:1754915. doi: 10.3389/fimmu.2026.1754915 (PMC13194116; doi:10.3389/fimmu.2026.1754915)
Supplement: Supplementary file 1 [file DataSheet1.zip › Supplementary Materials/Table 4. Results of risk factor analysis.docx]

**Table 4a.**Single factor meta-analysis

| Risk factors | The studies mentioned | heterogeneity | | OR/WMD (95%CI) | P | Egger |
| --- | --- | --- | --- | --- | --- | --- |
|  |  | I^2^(%) | P |  |  |  |
| Female | 12 | 81.9 | 0.000 | 1.47 (1.15, 1.89) | 0.002 | 0.717 |
| Age of onset | 11 | 89.7 | 0.000 | 1.09 (-0.29, 2.47) | 0.120 | 0.416 |
| Duration of RA (years) | 8 | 97.8 | 0.000 | -0.40 (-0.91, 0.10) | 0.120 | 0.280 |
| 28-joint disease activity score (DAS28) | 4 | 85.8 | 0.000 | -0.19 (-0.76, 0.38) | 0.517 | 0.345 |
| Duration of diabetes | 11 | 89.9 | 0.000 | 1.28 (-0.24, 2.79) | 0.099 | 0.439 |
| BMI | 3 | 0.0 | 0.744 | -0.32 (-0.79, 0.15) | 0.186 | 0.454 |
| ESR | 3 | 58.6 | 0.089 | -0.89 (-10.95, 9.17) | 0.863 | 0.869 |
| CRP | 3 | 77.7 | 0.011 | 0.42 (-0.15, 0.98) | 0.147 | 0.282 |
| Use of corticosteroids | 7 | 97.0 | 0.000 | 4.59 (1.92, 11.01) | 0.001 | 0.251 |
| Use of methotrexate | 10 | 98.0 | 0.000 | 1.92 (1.15, 3.19 ) | 0.012 | 0.198 |
| Use of hydroxychloroquine | 6 | 96.6 | 0.000 | 2.67 (1.24, 5.74) | 0.012 | 0.242 |
| Use of leflunomide | 6 | 95.2 | 0.000 | 2.02 (0.79, 5.16） | 0.140 | 0.282 |
| Use of tumor necrosis factor inhibitors | 5 | 89.0 | 0.000 | 1.48 (0.80, 2.75） | 0.212 | 0.839 |
| Use of sulfasalazine | 4 | 94.8 | 0.000 | 1.68 (0.86, 3.28） | 0.130 | 0.773 |
| ACPA (+) | 3 | 0.0 | 0.789 | 0.86 (0.50, 1.50） | 0.603 | 0.214 |
| History of diabetes mellitus | 9 | 90.1 | 0.000 | 1.37 (0.96, 1.96） | 0.088 | 0.412 |
| History of pulmonary disease | 7 | 90.7 | 0.000 | 1.27 (0.74, 2.17） | 0.381 | 0.059 |
| History of cancer | 6 | 90.9 | 0.000 | 1.59 (0.73, 3.49） | 0.244 | 0.264 |
| History of kidney disease | 6 | 78.2 | 0.000 | 2.05 (1.29, 3.27） | 0.003 | 0.071 |
| History of hypertension | 5 | 97.1 | 0.000 | 1.68 (1.12, 2.53) | 0.012 | 0.769 |
| History of liver disease | 4 | 76.2 | 0.006 | 1.38 (0.88, 2.18) | 0.162 | 0.255 |
| History of heart disease | 3 | 80.7 | 0.006 | 2.30 (1.17, 4.52) | 0.016 | 0.521 |

**Table 4b.**Multi-factor meta-analysis

| Risk factors | The studies mentioned | heterogeneity | | OR (95%CI) | P | Egger |
| --- | --- | --- | --- | --- | --- | --- |
|  |  | I^2^(%) | P |  |  |  |
| Corticosteroid dosage≥7.5mg/day | 5 | 48.4 | 0.101 | 2.16 (1.85, 2.53) | 0.000 | 0.501 |
| Use of corticosteroids | 5 | 0.0 | 0.730 | 1.42 (1.19, 1.69) | 0.000 | 0.872 |
| Use of tumor necrosis factor inhibitors | 3 | 0.0 | 0.704 | 1.94 (1.43, 2.63 ) | 0.000 | 0.851 |
| History of diabetes mellitus | 4 | 69.8 | 0.019 | 1.21 (0.99, 1.48) | 0.066 | 0.786 |
| Age | 5 | 92.3 | 0.000 | 1.12 (1.02, 1.22 ) | 0.012 | 0.132 |
| History of pulmonary disease | 3 | 65.7 | 0.054 | 1.42 (1.10, 1.83) | 0.007 | 0.070 |
| History of hypertension | 3 | 80.4 | 0.006 | 1.43 (1.15, 1.77) | 0.001 | 0.801 |
| History of kidney disease | 3 | 0.0 | 0.469 | 1.30 (1.04, 1.62) | 0.022 | 0.472 |
| Use of methotrexate | 3 | 0.0 | 0.373 | 1.68 (1.39, 2.02) | 0.000 | 0.747 |
